# Approach to vaccinating the pediatric solid organ transplant candidate and recipient

**DOI:** 10.3389/fped.2023.1271065

**Published:** 2023-11-08

**Authors:** Carol M. Kao, Marian G. Michaels

**Affiliations:** ^1^ Department of Pediatrics, Washington University in St. Louis, St. Louis, MO, United States; ^2^ Department of Pediatrics and Surgery, UPMC Children’s Hospital of Pittsburgh, Pittsburgh, PA, United States

**Keywords:** vaccines, immunizations, vaccine-preventable infections, solid organ transplant, pre-transplant evaluation

## Abstract

Solid organ transplantation (SOT) candidates and recipients are at increased risk for morbidity and mortality from vaccine-preventable infections. Children are at particular risk given that they may not have completed their primary immunization series at time of transplant or have acquired natural immunity to pathogens from community exposures. Multiple society guidelines exist for vaccination of SOT candidate and recipients, although challenges remain given limited safety and efficacy data available for pediatric SOT recipients, particularly for live-vaccines. After transplant, individual patient nuances regarding exposure risks and net state of immunosuppression will impact timing of immunizations. The purpose of this review is to provide readers with a concise, practical, expert-opinion on the approach to vaccinating the SOT candidate and recipient and to supplement existing guidelines. In addition, pediatric-specific knowledge gaps in the field and future research priorities will be highlighted.

## Introduction

Despite efforts to optimize childhood immunizations, vaccine-preventable infections remain a significant cause of morbidity and mortality for solid organ transplantation (SOT) candidates and recipients (1–3). Young children are at particular risk given that they may not yet have acquired natural immunity to pathogens nor had the opportunity to complete their primary immunization series at the time of transplant. In fact, pediatric SOT recipients are at 87 times increased risk for hospitalization from a vaccine-preventable infection in the first 5 years after transplant compared to the general population (1). The impact of a vaccine-preventable infection in SOT recipients is associated with prolonged hospitalization, increased healthcare costs, and increased risk for death or graft loss (4, 5). Rates of vaccine-preventable infections in this vulnerable population may be higher than previously estimated in light of declining worldwide childhood immunization coverage as a consequence of the COVID-19 pandemic and vaccine hesitancy (6). Recent outbreaks of previously eliminated infections, such as measles, are a reminder for ongoing vigilance (7).

Pediatric SOT candidates should ideally receive all age-appropriate vaccines prior to transplantation, given their attenuated immune response to vaccines post-transplant (8). The need for ongoing immunosuppression to prevent organ rejection, diminished immunogenicity to vaccines, and waning vaccine titers contribute to a heightened infectious risk in the post-transplant period (9). Family members and close contacts should also ensure they are up-to-date on their vaccines (10). Oftentimes the urgency of transplant in young children and infants precludes their ability to complete the primary vaccine series even using an accelerated vaccine schedule (11). Incomplete vaccines and annual influenza and COVID-19 vaccine can be administered post-transplantation based on timing and net state of immunosuppression, although certain criteria should be met before administering live vaccines (12).

Pre and post-SOT immunization guidelines and recommendations are available through infectious disease and transplant societies such as the Infectious Diseases Society of America and the American Society of Transplantation (AST) (12–14). However, rates of immunizations in SOT candidates and recipients remain sub-optimal. Less than 30% of pediatric SOT candidates are up-to-date at time of transplant based on the Centers for Disease Control and Prevention (CDC) childhood immunization schedules (15). Factors contributing to low immunization rates in transplant candidates include lack of coordination between multidisciplinary teams, less frequent care from primary care physicians, gaps in knowledge about optional timing and safety of pre-transplant immunizations, and lack of centralized immunization records (16). Given nuances in each patient scenario, there is not likely a “one size fits all” approach. The purpose of this review is to provide a concise, practical expert-opinion approach to vaccinating the SOT candidate and recipient and to highlight knowledge gaps and priorities for future research.

## Case 1

You are asked to see a 4-month-old boy with hypoplastic left heart syndrome who underwent Stage 1 palliative cardiac surgical repair. He presents to the Emergency Department with poor feeding, increased work of breathing, and is admitted to the hospital in acute heart failure. A ventricular assist device (VAD) is placed and he is listed status 1A for heart transplantation. His parents report he has received his first set of childhood immunizations at 2 months of age. What should be checked as part of his pre-transplant evaluation and what vaccines would you recommend?

### Pre-transplant vaccine recommendations

The pre-transplant screening visit is an opportunity to evaluate a candidate's risk for infection, including checking select vaccine serologies, and provide counseling about safe living after transplantation (10, 14, 17). The immunization history from the infant in Case 1 reveals that he has only received one set of routine vaccines 2 months ago, thus checking vaccine titers is not necessary, and he should receive routine vaccines now. If he is still waiting for a heart transplant in 4 weeks, the third set of primary childhood vaccines can be administered as part of the accelerated schedule (Table 1). In general, after review of records and serologic responses to certain vaccines, a vaccine roadmap should be developed and implemented as noted in Table 1 with additional considerations for asplenia or other conditions (Table 2).

**Table 1:** Pre and post-SOT vaccine schedule.

Vaccine	Type	Recommended before transplant	Recommended after transplant	Evaluation for serologic response	Minimum Age/Number of doses	Minimum interval between doses
Hepatitis B	Inactivated	Yes	Yes	Yes	Birth/2–3 doses	4–8 weeks
Diphtheria, tetanus, pertussis	Inactivated	Yes	Yes	No	6 weeks/4–5 doses	4 weeks to 6 months
*Haemophilus influenzae* b	Inactivated	Yes	Yes	No	6 weeks/1–4 doses	4–8 weeks
Polio	Inactivated	Yes	Yes	No	6 weeks/3–4 doses	4 weeks to 6 months
*Streptococcus pneumonia*	Inactivated	Yes	Yes	No	6 weeks/1–4 doses	4–8 weeks
Meningococcus serogroup ACWY	Inactivated	Yes	Yes	No	2 months/2–4 doses	8 weeks to 6 months
Meningococcus serogroup B	Inactivated	Yes in specific cases	Yes	No	2 months to 10 years/2 doses	4 weeks to 6 months
Hepatitis A	Inactivated	Yes	Yes	Yes	6 months/1–3 doses	6 months
HPV	Inactivated	Yes	Yes	No	9 years/2–3 doses	4 weeks to 5 months
Influenza	Inactivated	Yes	Yes	No	6 months/2 doses^a^	4 weeks
SARS-CoV-2	mRNA	Yes	Yes	No	6 months	4 weeks
Varicella	Live attenuated	Yes	Not routine/can be considered for specific patients	Yes	6 months/2–3 doses	4 weeks
Measles, mumps, rubella	Live attenuated	Yes^b^	Not routine/can be considered for specific patients	Yes	6 months/2–3 doses	4 weeks
Rotavirus	Live attenuated	Yes^b^	Not routinely	No	6 weeks/2–3 doses	4 weeks

Table adapted from CDC child and adolescent immunization schedule by age, recommendations for ages 18 years or younger, 2023.

^a^ Two doses of influenza vaccine for children 6 months to 8 years who have received fewer than 2 vaccines in any previous year or whose influenza vaccination history is unknown.

^b^ Live attenuated vaccines MMR and varicella dosing pre-transplant may be impacted by passive antibody presence from mother or blood products and anticipated time of accepting organs with ideally having 3–4 weeks between vaccination and accepting an organ for MMR. In addition, after transplant specific patients could be considered for vaccination if on stable low immunosuppression.

**Table 2:** Additional vaccines for asplenic or hyposplenic SOT candidate and recipients.

Pathogen	Vaccine	Minimum age	Minimum interval between doses	Total number of doses
Pneumococcus	PCV15	6 weeks	4 weeks to 6 months	1–4
	PCV20	6 weeks	4 weeks to 6 months	1–4
	PPSV23^a^	2 years	8 weeks after PCV, 2nd dose 5 years	2
Meningococcus	MenACWY-CRM (Menveo)	2 months	8 weeks to 6 months	2–4
	MenACWY-D (Menactra)	9 months	8–12 weeks	2
	MenACWY-TT (MenQuadfi)	2 years	8 weeks	2
	MenB-4C (Bexsero)^b^	2 months to 10 years	4 weeks	2
	MenB-FHbp (Trumenba)^b^	2 months to 10 years	1–6 months	2–3
*Haemophilus influenzae*	Hib	6 weeks	4–8 weeks	1–4

^a^ PPSV23 not required after PCV20.

^b^ MenB vaccines are approved for use in infants 2 months and older in the European Union, UK and Canada.

For vaccinated children, serology can be checked for Hepatitis B (HBV), Hepatitis A (HAV), varicella (VZV), and measles/mumps/rubella (MMR) (14). To have maximal benefit, inactivated vaccines should be given at least 2 weeks prior to transplant. It is recommended to have at least 4 weeks before transplant for safety of administering live vaccines (14). Given that the infant in Case 1 is only 4 months of age and requires a VAD, he likely has passive antibody that would render live virus vaccines, such as MMR and varicella, useless; likewise, he is also listed status 1A for heart transplant with the potential for transplant to occur within 4 weeks, and thus live vaccines should not be given.

Family members and close contacts of the SOT candidate should ensure they are up-to-date on their immunizations as well, including seasonal influenza and COVID-19 vaccines. The latter two vaccines are of critical importance in infants too young to be vaccinated themselves, such as in Case 1. Live vaccines are not contraindicated in close contacts with the exception of the smallpox and oral polio vaccines; the latter is not available in the United States. Contact precautions and hand hygiene should be used if the rotavirus vaccine or live-attenuated influenza vaccines are given. Likewise, if a rash develops after varicella vaccine, direct contact with the rash should be avoided until complete crusting occurs (18). Household pets should also have annual check-ups to ensure they are healthy and fully immunized (14). Vaccine specific recommendations are outlined below.

### Hepatitis B

Hepatitis B (HBV) infection is highly prevalent worldwide and universal vaccination is recommended for all infants, children, and adolescents to prevent progressive liver damage (14, 19). Immunity against HBV should be confirmed prior to transplant (anti-HBs >10 IU/ml) as antibody titers can wane, particularly in immunocompromised individuals or those receiving chronic dialysis (14, 20). Assessing HBV immunity became part of the Organ Procurement and Transplantation Network (OPTN) policy in 2021. Future analysis will help assess vaccine efficacy, particularly in cases of HBV core antibody positive donors. In a retrospective study of pediatric SOT candidates, approximately half had indeterminate, non-reactive, or unavailable HBV surface antibody titers, even amongst those who had completed the primary vaccine series (20). Documentation of immunity is of particular importance in SOT recipients as donor-derived infection has been reported and adequate titers has been shown to prevent *de novo* HBV infection post-transplant (21). In non-immune individuals (anti-HBs <10 IU/ml), revaccination can be done with the complete series, using high-dose (40 µg) vaccine, or with giving one dose and re-checking anti-HBs (14). An adjuvanted two-dose HBV vaccine (HepB-CpG) with increased immunogenicity compared to existing vaccine formulations is available for individuals older than 18 years of age (22).

### Diphtheria, tetanus, and pertussis

All pediatric SOT candidates and recipients should receive diphtheria, tetanus and pertussis containing vaccines using an acellular pertussis component (DTaP or TdaP) (14). Infants and children should receive 5 doses DTaP vaccines with minimal intervals specified in Table 1. Combination vaccines are available and can be used to reduce the number of overall injections. A single booster dose of Tdap should be administered to adolescents and Tdap should be given every 10 years throughout life for ongoing tetanus prophylaxis (19).

### *Haemophilus influenzae* type B (Hib)

*Haemophilus influenza* type B (Hib) infections have declined dramatically since the introduction of the Hib vaccine (19). Immunocompromised individuals particularly those with asplenia or impaired splenic function are at particular risk for invasive infection (23). After completion of the series vaccine titers are not routinely checked.

### Polio

Inactivated polio vaccine (IPV) should be administered to all SOT recipients and can be safely administered post-transplant, with minimal intervals specified in Table 1 (19). Oral polio vaccine stopped being used in the United States since 2000 given risk for vaccine-associated paralytic poliomyelitis in vaccine recipients or close contacts. Being up to date on IPV is underscored with the resurgence of polio in the United States and other countries in unvaccinated people (24). After completion of the series vaccine titers are not routinely checked.

### Streptococcus pneumoniae

Invasive pneumococcal disease (IPD) causes significant morbidity and mortality in children and can lead to bacteremia, pneumonia, endocarditis or meningitis (25). SOT recipients are at significantly higher risk for IPD, particularly those with asplenia or infants heart transplant recipients (3). Two types of pneumococcal vaccines are available, protein-conjugated vaccines and the 23-valent polysaccharide vaccine used in certain high-risk individuals. In June 2022, the CDC recommended the use of 15-valent pneumococcal conjugate vaccine (PCV15) in children; PCV20 was subsequently approved in June 2023 (25). PCV15 contains two additional serotypes (22F, 33F) and PCV20 also contains (8, 10A, 11A, 12F and 15B). In a Phase III trial (NCT03921424) of PCV15 in children infected with Human Immunodeficiency Virus (HIV), PCV15 was more immunogenic for 8 shared serotypes compared to PCV13, although specific data in SOT recipients is lacking (25). Current recommendations are for the use of either PCV15 or PCV20 for all infants and children as the primary series (Table 3). For SOT recipients who have not received any dose of PCV13, PCV15, or PCV20, a single dose of PCV15 or PCV20 is recommended ≥8 weeks after the most recent dose of pneumococcal vaccine (26). In children who receive PCV20, additional administration of PPSV23 is not needed.

**Table 3:** Updated recommendations for pneumococcal vaccination with PCV15 or PCV20 in healthy children and those with high-risk conditions.

Age	Previous dose	PCV15/PCV20 regimen
<2 years of age	Unvaccinated	4 doses of PCV15 or PCV20
2–5 years of age	Unvaccinated or incompletely vaccinated (<3 doses)	1 additional dose ≥8 weeks after most recent
5–18 years of age	Unvaccinated or incomplete	No additional dose
Children with high-risk conditions^a^		
<2 years of age	Unvaccinated	4 doses of PCV15 or PCV20
2–5 years of age	Unvaccinated or <3 doses by 2 years of age	2 doses, ≥8 weeks apart between each dose
	3 doses	1 additional dose ≥8 weeks after most recent
6–18 years of age	Unvaccinated or incomplete	1 dose PCV20 ≥8 weeks after most recent 1 dose PCV15 ≥8 weeks and 1 dose PPSV23 ≥8 weeks after if not previously given
	Fully vaccinated	If completed with PCV20, no additional doses If completed with PCV13/15, 1 dose PCV20 ≥8 weeks after most recent dose or PPSV23 using previously recommended schedule

^a^ Chronic heart disease, chronic kidney disease, chronic liver disease, chronic lung disease, diabetes mellitus, cerebrospinal fluid leak, cochlear implant, maintenance dialysis or nephrotic syndrome, congenital or acquired asplenia, or splenic dysfunction, congenital or acquired immunodeficiency, diseases and conditions treated with immunosuppressive drugs or radiation therapy, HIV infection, sickle cell disease or other hemoglobinopathies, solid organ transplantation.

### Meningococcal

Meningococcal disease caused by *Neisseria meningitidis* can cause bacteremia, meningitis or invasive infection particularly in high-risk individuals (27). Three quadrivalent conjugate vaccines against serogroups A, C, W, and Y (MenACWY) and two serogroup B meningococcal vaccines (MenB) are available. Vaccination with MenACWY is recommended for adolescents 11 or 12 years of age and infants older than 2 months at increased risk, such as those with complement deficiency, receipt of complement inhibitor, anatomic or functional asplenia, travel to a high risk area, or those with HIV (27). Vaccine should ideally be given at least 2 weeks prior to splenectomy or receipt of complement inhibitor. In the United States, MenB vaccine is recommended starting at 10 years of age in individuals at high-risk for meningococcal infection with a booster dose for those who remain at increased risk. Canadian and European Guidelines start as early as 2 months of age. For asplenic individuals, booster doses of MenACWY should be given every 5 years and MenB every 2–3 years (27). Transplant recipients receiving terminal complement inhibitors to treat antibody-mediated rejection are at particular risk for meningococcal disease and should be vaccinated prior to treatment.

### Hepatitis A

Hepatitis A virus (HAV) is a communicable disease transmitted fecal-orally (28). Periodic outbreaks occur typically from contaminated foods. Immunocompromised individuals and those with underlying liver disease are at risk for severe infection and fulminant hepatitis. HAV vaccine should be administered to all SOT candidates and recipients, particularly liver transplant candidates (14, 28). Serologic response to HAV should be checked to ensure seroconversion (14).

### Human papillomavirus

Human papillomavirus (HPV) is a common sexually transmitted infection which can lead to the development of cervical, anogenital, and oropharyngeal cancers. SOT recipients have higher rates of HPV cancers compared to the general population (29). Vaccination against HPV has been routinely recommended since 2006 for females and 2011 for males (19). Two or three-doses of HPV vaccine are recommended starting as early as 9 years of age. While the vaccine is safe to be given post-transplant, immunogenicity may be reduced, particularly in lung transplant recipients likely related to degree of immunosuppression (30). Because of this, efforts should be made to optimize HPV vaccine pre-transplant.

### Influenza

Influenza causes severe infection in immunocompromised individuals and annual vaccination is strongly recommended for SOT candidate and recipients 9 months and older and their close contacts (31). Live attenuated influenza vaccine should not be given to SOT recipients given the theoretical concern for viral replication. Although current recommendations are the same as those for the general public, SOT recipients likely have decreased immune response to the influenza vaccine and may benefit from alternative strategies, such as high-dose influenza vaccine or an additional booster dose (14, 32, 33). The optimal timing of influenza vaccine administration post-transplant remains a topic of interest. Current recommendations are for the influenza vaccine to be given 3 months post-transplant, although it is often given as early as 1 month post-transplant when there is community spread.

### SARS-CoV-2

A number of COVID-19 vaccines are approved and the most up-to-date recommendations can be found on the CDC and AST websites (34, 35). Vaccination against SARS-CoV-2 is strongly recommended for everyone 6 months and older, and ideally should be completed prior to transplantation. However, it can be given as early as 1–3 months post-transplant depending on community circulation. Three doses of an mRNA vaccine is recommended as the primary series for SOT recipients given decreased humoral and cellular immune response with two doses, although some SOT recipients will fail to mount any detectable humoral response even after booster doses (36). Current recommendations include age-appropriate bivalent booster dose to be given at least 2 months after the last dose of vaccine, although the CDC website should be consulted for the most up to-date information given the evolving nature of the COVID-19 pandemic.

### Varicella

Varicella vaccine (VZV) is a live-attenuated vaccine routinely administered as a 2-dose series at 12–15 months and 4–6 years of age, although the second dose can be given as early as 4 weeks after the first. While efficacy is not assured, infants can receive a dose at 6 months with a second and third dose given if transplant has not occurred by 12 months of age (13, 14). Passive antibody from blood products may interfere with efficacy and timing of vaccination should be based on CDC recommended time intervals, unless serology is negative (12). Serology should be checked prior to transplant. Efforts should be made to administer both doses of VZV vaccine prior to transplant given concerns for safety of live vaccine administration post-transplant until immunosuppression can be lessened. If vaccine is given and an organ becomes available within 4 weeks, acyclovir can be administered to prevent vaccine virus infection. After SOT, VZV vaccine administration can be considered in select individuals (Table 4) (12).

**Table 4:** Considerations for live vaccine eligibility after kidney or liver transplant.

Defer vaccine	• Clinically unwell • Cardiac, lung, multivisceral transplant recipient^a^ • High-level immune suppression • Current rejection • Use of novel biologic agents other than those listed below • Receipt of ATG <1 year • Receipt of Alemtuzumab <2 years • Receipt of Rituximab <1 year
Vaccinate with caution	• Current use of mycophenolate mofetil/sodium • Persistently elevated EBV viral loads • Liver transplant recipients undergoing immune suppression withdrawal with goal of cessation or those with “functional tolerance”
Proceed with vaccine	Should meet all of the following criteria • Clinically well and do not need any of the contraindications listed above • At least 1 year post-transplant and/or 2 months post-rejection • Steroid dose (prednisone equivalent) <2 mg/kg/day or total cumulative <20 mg/day • Tacrolimus <8 ng/ml for two consecutive readings • Cyclosporine <100 ng/ml for two consecutive readings • ALC >1,500 for children ≤6 years and >1,000 cells/µl for children >6 years of age • CD4 >700 cells/µl for children ≤6 years and >500 cells/µl for children >6 years of age • Normal total serum IgG for age

^a^ Some experts have given live vaccines to recipients of organs other than liver and kidney with caution and close follow-up.

### Measles, mumps and rubella

The live-attenuated measles, mumps, rubella (MMR) vaccine is typically given as a two-dose series at 12–15 months and 4–6 years of age. Similar to VZV vaccine, the second dose can be given with only a 4-week interval and for infants a dose can be given as early as 6 months of age. This should strongly be considered in infants not anticipated to be transplanted within 4 weeks, as post-transplant administration is generally contraindicated or may be considerably delayed (12). Early administration may result in reduced immunogenicity from immature immune responses and interfering maternal antibodies, and thus the two-dose series should be re-initiated if transplant has not occurred by 12 months of age and is not anticipated to occur within 4 weeks. Vaccine should be delayed an appropriate interval if blood products or immunoglobulin has been given due to interference with immune response. MMR serology should be checked prior to transplant.

## Case 2

You are called about a 3-year-old girl who is now 2 years post-liver transplant without concerns for rejection. She remains on tacrolimus. Parents are concerned she will start preschool in the fall and may be exposed to additional germs from classmates. What vaccine recommendations can you provide to parents?

### Post-transplant vaccine recommendations

In general, inactivated vaccines are safe to administer 3–6 months post-transplant, although the optimal timing depends on the individual circumstances and net immunosuppression (14). If the individual in case 2 were not up-to-date on any of her primary vaccine series, it would be important to administer them in an expedited schedule at this time (Table 1). Live vaccines have not traditionally been recommended post-transplant, however can be considered in select individuals who meet clinical and immunologic criteria listed in Table 4. There is increasing evidence for the safety and immunogenicity of live vaccines given after SOT and expert guidelines have been developed for select kidney and liver transplant recipients (12). Live vaccines can considered in individuals who are clinically well, at least 1 year post-transplant without concern for rejection, on low-level immune suppression, and meet minimum immune criteria (12). If the individual in case 2 had normal immune parameters, Varicella and then MMR vaccines could be offered to the family. Results from a multicenter study containing 281 pediatric kidney and liver transplant recipients who received Varicella or MMR vaccines found live vaccines to be both safe and immunogenic (37).

### Research gaps

While significant advances have been made in the field, uncertainty remains about optimal timing, durability of immune responses post-transplant and need for booster doses, and the safety and immunogenicity of new vaccines (38). In addition, the approach to live vaccines both pre and post-transplant is variable across institutions and warrants further study. In a small case series of 5 pediatric heart and liver recipients who received live vaccines 8–21 days prior to transplant, none developed vaccine-related adverse events or concern for viral illness (39). This suggests that a shorter interval may be safe, and transplant should not be delayed if live vaccines are inadvertently administered; although, it is important to note the sample size was small and four of the five individuals received post-exposure prophylaxis with IVIG and antiviral therapy.

There are a number of novel vaccine candidates in various stages of development, including candidates against respiratory syncytial virus (RSV) and cytomegalovirus. The first vaccine against RSV was recently approved by the US Food and Drug Administration for use in adults 60 years and older. RSV is a common cause of respiratory viral infections in infants and children and significant cause of severe infection in SOT recipients. There are a number of RSV vaccine candidates in late stages of clinical trials, and some already approved for pregnant women and the elderly with the hope for future trials in immunocompromised individuals.

## Conclusions

Vaccine preventable infections remain a significant problem in pediatric SOT candidates and recipients. Efforts to optimize pre and post-transplant vaccines should be a priority for all clinicians taking care of SOT patients in order to optimize post-transplant outcomes.

## Data Availability

The original contributions presented in the study are included in the article/Supplementary Material, further inquiries can be directed to the corresponding author.
